# Acute Colonic Pseudo-Obstruction in a Patient With COVID-19 Pneumonia: A Case Report

**DOI:** 10.7759/cureus.36251

**Published:** 2023-03-16

**Authors:** Ahmad Mahdi, Aastha Bharwad, Mahmoud Mahdi, Kyle Rowe

**Affiliations:** 1 Internal Medicine, University of Kansas School of Medicine-Wichita, Wichita, USA

**Keywords:** abdominal radiology, treatment choices, kub, covid 19, acute colonic pseudo-obstruction

## Abstract

Coronavirus disease (COVID-19) is primarily a respiratory illness caused by the severe acute respiratory syndrome coronavirus 2 (SARS-CoV-2) virus. However, the disease is also known to cause a range of extrapulmonary manifestations, including gastrointestinal (GI) symptoms such as nausea, vomiting, and diarrhea. The exact mechanisms by which the virus causes extrapulmonary manifestations are not fully understood, but it is theorized that the virus can enter cells in other organs including the GI tract, through the angiotensin-converting enzyme 2 (ACE2) receptor. This can result in inflammation and damage to the affected organs. In rare cases, COVID-19 can also cause acute colonic pseudo-obstruction (ACPO), a condition characterized by symptoms of bowel obstruction but without a physical obstruction present. Acute colonic pseudo-obstruction is a serious and potentially life-threatening complication of COVID-19 that requires prompt recognition and treatment to prevent further complications such as bowel ischemia and perforation. We hereby present a case report of a patient with COVID-19 pneumonia developing ACPO and discuss the suggested pathophysiology, diagnostic approach, and treatment options.

## Introduction

The SARS-CoV-2 virus has caused a global pandemic, infecting millions of individuals worldwide. Coronavirus disease caused by SARS-CoV-2 typically presents with respiratory symptoms such as fever, cough, and shortness of breath [1,2]. However, numerous studies have reported a variety of extra-pulmonary symptoms and complications of COVID-19, including gastrointestinal (GI), cardiovascular, and neurological symptoms. Among the extra-pulmonary symptoms associated with COVID-19, GI symptoms have become increasingly evident in newer strains of SARS-CoV-2 [3].

Gastrointestinal symptoms reported in COVID-19 patients include anorexia, diarrhea, nausea/vomiting, and abdominal pain, and in some cases, GI symptoms may be the only presenting sign of the disease [4]. A systematic review of studies reporting GI symptoms in confirmed COVID-19 patients noted an overall pooled prevalence of 18%, with diarrhea, nausea/vomiting, and abdominal pain being the most common manifestations [5]. Acute colonic pseudo-obstruction (ACPO), also known as Ogilvie's syndrome, is a rare but potentially life-threatening complication that has been reported in a few cases of COVID-19 [6]. It is a clinical condition characterized by acute large bowel obstruction without mechanical blockage [7].

We present a case of COVID-19 pneumonia complicated by ACPO and explore the possible underlying mechanisms, diagnostic approaches, and potential treatments.

## Case presentation

A 71-year-old male patient with a past medical history of hypertension and obesity presented with shortness of breath and cough for four days prior to presentation. On physical examination, he was found to be afebrile, confused, hypoxic with an oxygen saturation of 88% on room air, tachycardic with a pulse of 108 beats per minute, and tachypneic with a respiratory rate of 22 breaths per minute. He had normal air entry bilaterally on chest auscultation and a soft yet distended abdomen. Supplemental oxygen was provided, and a positive COVID-19 polymerase chain reaction (PCR) confirmed his infection.

**Figure 1:**
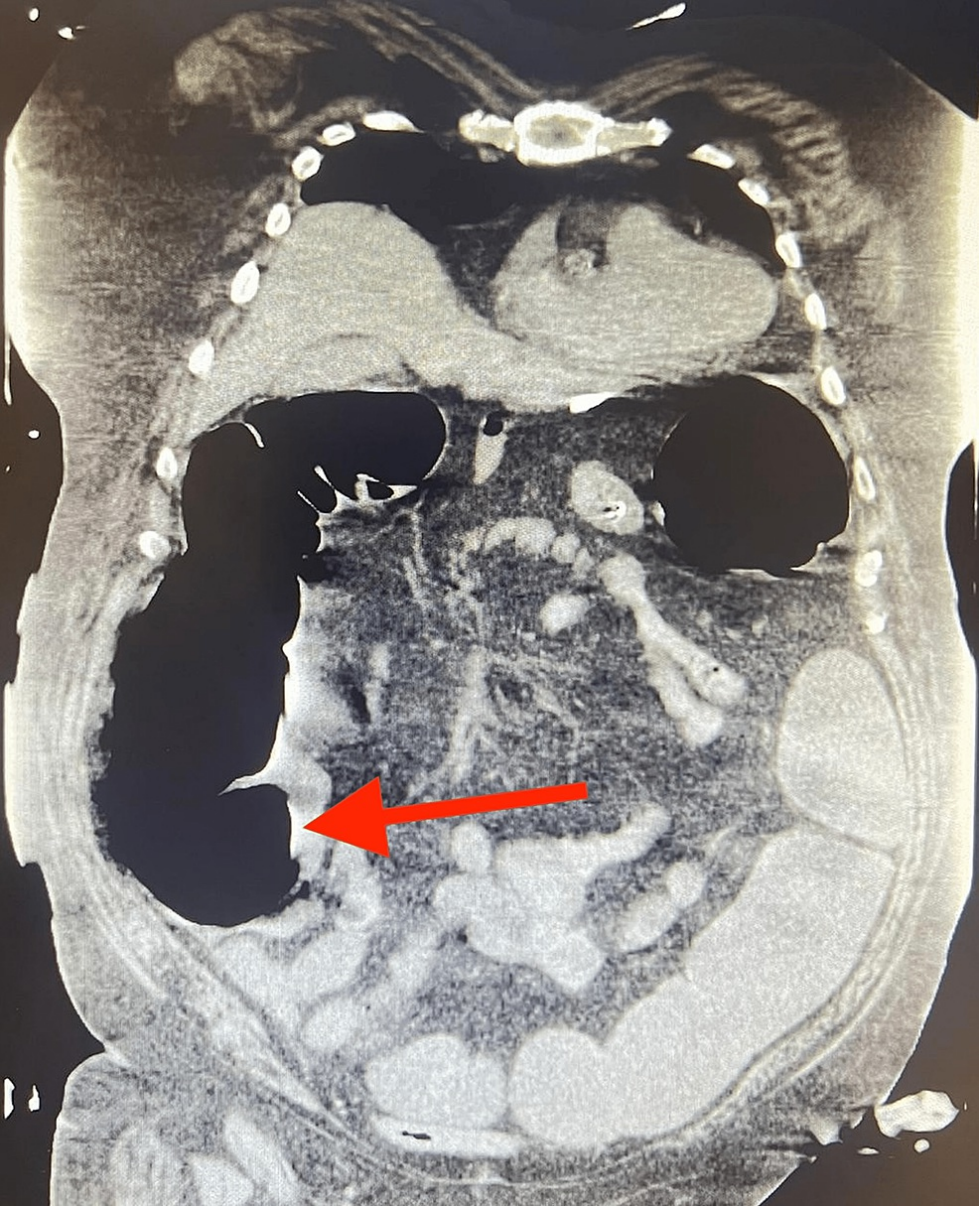
CT of the abdomen and pelvis without contrast showing dilated large bowel segments without transition point or obstructing lesion

On admission, WBC was elevated at 22.3 cells per cubic millimeter with neutrophilic predominance; sodium and potassium were low at 121 mEq/L and 3 mEq/L, respectively; and serum creatinine was elevated at 4.13 mg/dL. Chest X-ray revealed bibasilar atelectasis with a suspected infiltrate in the right base. The kidney, ureter, bladder (KUB) X-ray revealed gaseous distention of the small bowel and colon, most likely due to ileus. The colon measured up to 11 cm at the level of the cecum (Figure 1). The patient was started on intravenous dexamethasone 6 mg daily for 10 days for COVID-19 treatment, ceftriaxone 1 gram/day for five days, and azithromycin 500 mg/day for three days for suspected superimposed community-acquired pneumonia. Sodium and potassium levels were corrected, other electrolytes were maintained within normal range, and lactated Ringer's solution was given.

Over the course of hospitalization, the patient’s colonic distension remained persistent despite the correction of electrolytes and careful management of all potential causes of ileus such as avoiding causative medications. Serial abdominal X-rays were performed to monitor the abdominal ileus, and management was conservative. A nasogastric tube was inserted to aid in bowel rest. The patient soon began experiencing respiratory distress secondary to the restrictive effect of the ileus on the diaphragmatic movements and shallow breathing, leading to intubation. Neostigmine was contraindicated given his systolic blood pressure was less than 90 mmHg. No other intestinal stimulant was used due to the risk of bowel rupture. A decompressive colonoscopy was performed twice during the intubation period for diagnostic and therapeutic purposes. However, the distension was only temporarily relieved on every occasion. His persistent colonic distension required him to be re-intubated for two days before extubating him again. Eventually, the patient was diagnosed with refractory and persistent acute colonic pseudo-obstruction secondary to COVID-19 pneumonia. The patient was eventually discharged to a long-term acute care facility where he passed away due to respiratory failure.

## Discussion

Acute colonic pseudo-obstruction is an infrequently reported complication associated with COVID-19 [6]. As the COVID-19 pandemic evolves, studies have found that extra-pulmonary symptoms and complications have been becoming more evident in newer strains of the virus [3]. Proposed theories for the pathophysiology of ACPO in COVID-19 patients suggest multiple mechanisms.

First, the ACE-2 receptor, the functional host receptor for the SARS-CoV-2 virus, has strong expressions on the brush border of the small and large intestinal mucosa. This may mediate the invasion and amplification of the virus with GI inflammation [8]. Second, COVID-19 is associated with inflammatory coagulopathy with reported cases of acute bowel wall ischemia, pneumatosis intestinalis, and colonic perforation due to microangiopathic thromboembolism and endothelial lining dysfunction. These effects may impair motility [9]. Finally, the SARS-COV-2 virus is known to have neurotropic potential, and could directly invade the myenteric plexus, resulting in loss of parasympathetic spinal control of bowel movement and manifesting as ACPO. Early recognition of ACPO is vital to implement timely management and deter lethal consequences such as bowel perforation [10].

The signs and symptoms of ACPO can be nonspecific and may overlap with other conditions, making the diagnosis challenging. Acute colonic pseudo-obstruction is characterized by abdominal distension, pain, nausea, vomiting, and obstipation, which can mimic a mechanical bowel obstruction. However, unlike mechanical obstruction, there are no signs of bowel wall edema or obstruction in imaging studies [11]. Diagnostic testing for ACPO includes abdominal radiography, which shows dilatation of the colon without signs of mechanical obstruction [12]. The CT scan is also helpful in differentiating ACPO from a mechanical obstruction by showing a dilated colon without a transition point, pneumatosis intestinalis, or colonic wall thickening, which can be seen in mechanical obstruction albeit not specific to it [13].

Treatment for ACPO aims to relieve colonic distension, prevent bowel ischemia and necrosis, and reduce the risk of perforation. Conservative management includes supportive measures such as nasogastric decompression, rectal tube insertion, and fluid and electrolyte management [14]. Medications such as neostigmine, a cholinergic agent, can be used to stimulate bowel motility and reduce colonic distension. Other prokinetic agents include erythromycin, metoclopramide, and cisapride [14,15]. In refractory cases, endoscopic decompression or surgical intervention may be necessary to relieve colonic obstruction and prevent bowel ischemia [16].

## Conclusions

Acute colonic pseudo-obstruction is a rare but potentially serious complication in COVID-19 patients. Abdominal distension, pain, nausea, vomiting, and obstipation are typical symptoms, and abdominal radiography and CT scan are diagnostic modalities. Conservative management with supportive measures, medications, and endoscopic or surgical intervention in refractory cases can be effective treatment options. Early recognition and management are crucial to prevent complications such as bowel ischemia and perforation.
